# Broadband Absorption Tailoring of SiO_2_/Cu/ITO Arrays Based on Hybrid Coupled Resonance Mode

**DOI:** 10.3390/nano9060852

**Published:** 2019-06-04

**Authors:** Jiqing Lian, Dawei Zhang, Ruijin Hong, Tingzhen Yan, Taiguo Lv, Daohua Zhang

**Affiliations:** 1 Engineering Research Center of Optical Instrument and System, Ministry of Education and Shanghai Key Lab of Modern Optical System, University of Shanghai for Science and Technology, Shanghai 200093, China; LianJiqing1990@163.com (J.L.); rjhongcn@163.com (R.H.); tzyancn@163.com (T.Y.); lvtaiguo@lcu.edu.cn (T.L.); 2 School of Electrical and Electronic Engineering, Nanyang Technological University, Singapore 639798, Singapore; edhzhang@ntu.edu.sg

**Keywords:** SiO_2_/Cu/ITO arrays, ITO thin films, ENZ mode, hybrid coupled resonance, FDTD, Macleod simulation, spectral tailoring

## Abstract

Sub-wavelength artificial photonic structures can be introduced to tailor and modulate the spectrum of materials, thus expanding the optical applications of these materials. On the basis of SiO_2_/Cu/ITO arrays, a hybrid coupled resonance (HCR) mechanism, including the epsilon-near-zero (ENZ) mode of ITO, local surface plasmon resonance (LSPR) mode and the microstructural gap resonance (GR) mode, was proposed and researched by systematically regulating the array period and layer thickness. The optical absorptions of the arrays were simulated under different conditions by the finite-difference time-domain (FDTD) method. ITO films were prepared and characterized to verify the existence of ENZ mode and Mie theory was used to describe the LSPR mode. The cross-sectional electric field distribution was analyzed while SiO_2_/Cu/ITO multilayers were also fabricated, of which absorption was measured and calculated by Macleod simulation to prove the existence of GR and LSPR mode. Finally, the broad-band tailoring of optical absorption peaks from 673 nm to 1873 nm with the intensities from 1.8 to 0.41 was realized, which expands the applications of ITO-based plasmonic metamaterials in the near infrared (NIR) region.

## 1. Introduction

Selective spectral tailoring and manipulation based on sub-wavelength artificial photonic structures have been widely used in many fields such as new energy sources, sensors, spectral detection imaging, nano-scale photothermical conversion and transfer [1,2,3]. In general, however, the interaction between light and matter is corruptible. The metamaterial structure based on surface plasmon polaritons (SPPs) can effectively control the transmission of super-diffraction limit light, and also converge and amplify the electromagnetic energy [4]. Moreover, selective scattering and absorption of light at different frequencies can be obtained by LSPR effect generated in metallic particles [5,6,7,8]. Cu is an important metal because of its high conductivity and thermal conductivity [9,10]. Although its LSPR effect lags behind that of gold and silver [11,12,13,14] and its surface is easily oxidized, the core-shell structure [15,16] can be formed through its oxide layer (Cu_2_O), which will stabilize Cu nanoparticles without weakening the LSPR effect. In addition, Cu has cost advantages compared to Ag and Au [17].

However, metal plasmonic materials have large losses in optical and communication bands [18]. On the one hand, researchers reduce the optical loss by introducing metal/dielectric interface coupling [19]. On the other hand, alternative plasmonic materials have been constantly searched for. The optical loss of ITO in the NIR region is lower than that of Ag [20,21]. Moreover, the carrier concentration can be flexibly tuned, and it has been proved to be good alternative to noble metals [22]. In 2018, ITO thin layer was deposited on top of In-Ga-Zn-O to inhibit the migration and diffusion of copper which improved the performance of transistors [23]. Therefore, ITO layer can not only protect the inner film from damage, but also effectively prevent the diffusion of the metal layer. In addition, the ITO ENZ characteristics can be achieved in a specified band, which enhances the interaction between ITO materials and light near the ENZ wavelength, producing a series of novel nonlinear optical properties [24]. What’s more, the ENZ mode greatly broadens the application of ITO in non-linear optics, such as all-optical modulation, perfect absorber, waveguide modulator and ultra-fast all-optical switch [25,26,27,28,29,30]. The tunability of ITO ENZ wavelength has been confirmed in our previous work [31].

In addition, the patterning of TCO-based metamaterials has reached nanometer scale [32,33,34]. The GR mode could be introduced by ITO-based metamaterials [35,36,37], which could enhance the local optical coupling strength and tune the wavelength range of plasma resonance [38,39,40]. The coupling of three plasma polaritons (SPs) was analysed based on the air/Ag/dielectric double dipole structure, which included SPs excitation by plane wave, dipole and local surface plasmon (LSP). It was found that the coupling of SPP and LSP induced a red shift when the thickness of the Ag film was reduced [41]. Wang et al. analyzed the overlap of electric dipole coupling and magnetic dipole plasma resonance between adjacent elements in metal/dielectric/metal (M/D/M) sandwich arrays [42].

Considering the non-linear ENZ characteristics of TCO materials, Feng et al. found that the interface coupling between metal and ENZ materials promoted the rapid propagation of plasma waves along the ENZ film [43]. Recently, a 240 nm wide and perfect (more than 98%) flat-top absorption was achieved by adding a 12 nm ITO layer into the patterned Au nanodisk and SiO_2_ dielectric layer with 1550 nm as the central wavelength. It was the strong coupling effect between the GP mode and ENZ mode in the super-surface that led to the broadband and perfect resonance absorption [35,44].

Based on ENZ mode (λ_ENZ_), LSPR mode (λ_LSPR_) of metal Cu and ITO and GP mode (λ_GAP_) of microstructures, the mechanism of broad-spectrum absorption regulation of SiO_2_/Cu/ITO microstructures was studied in this paper. A Cu layer was introduced to simulate the LSPR effect in the array while ITO was chosen for the existence and tunability of the ENZ mode. Firstly, the absorption of SiO_2_/Cu, SiO_2_/ITO and SiO_2_/Cu/ITO arrays were analyzed by FDTD simulation to identify the array model. Then, selective tailoring and regulation of SiO_2_/Cu/ITO arrays from visible to NIR bands were realized by changing the array period and the thickness of Cu or ITO layers. ITO ENZ mode was validated through ITO thin films prepared by electron beam evaporation, by which SiO_2_/Cu/ITO multilayers were also prepared. The absorptions were measured and calculated through the Macleod method. What’s more, the evolution of LSPR mode and GR mode was due to the cross-section electric field distribution. Finally, the HCR mode (λ_HCR_) in SiO_2_/Cu/ITO arrays was confirmed to exist.

## 2. Methods and Model Design

**Figure 1:**
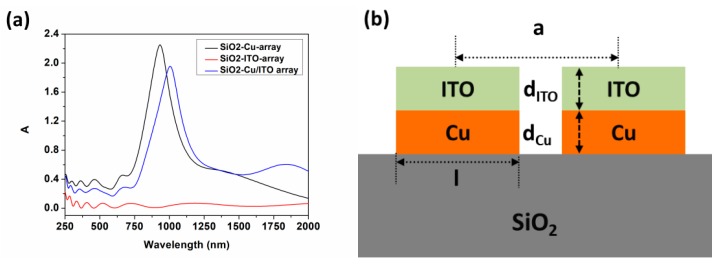
(**a**) The optical absorptions of the arrays; (**b**) The schematic diagram of the model.

The simulation software, materials and characterization methods used in this study are presented in the Supplementary Materials Table S1. Here, optical absorption of SiO_2_/ITO arrays, SiO_2_/Cu arrays and SiO_2_/Cu/ITO arrays were compared at the same period (110 nm) and thickness (100 nm × 100 nm × 10 nm) after simulation. The absorption was calculated by A = 1 − T − R. As can be seen from Figure 1, there are no noticeable absorption peaks in the visible to NIR range for ITO arrays and the overall absorption values are in small bands. However, the single Cu array shows strong absorption peaks only in the short near infrared waveband (S-NIR, 780–1100 nm).

SiO_2_/Cu/ITO arrays can simultaneously satisfy the coupling resonance absorptions in S-NIR and middle near infrared wave band (M-NIR, 1100-2000 nm), which make it possible to achieve broad-spectrum absorption regulation in NIR. As a result, the array model we chose is indicated above. Cu layer was inserted into the quartz substrate (SiO_2_) and ITO layer. *d_ITO_* and *d_Cu_* represent the thickness of ITO and Cu respectively. The length and width of the array unit are 100 nm and the period is expressed in *a*. During the simulating, *a*, *d_ITO_* and *d_Cu_* are variables.

## 3. Results and Discussion

### 3.1. Modulation of Absorption in Different Periods

**Figure 2:**
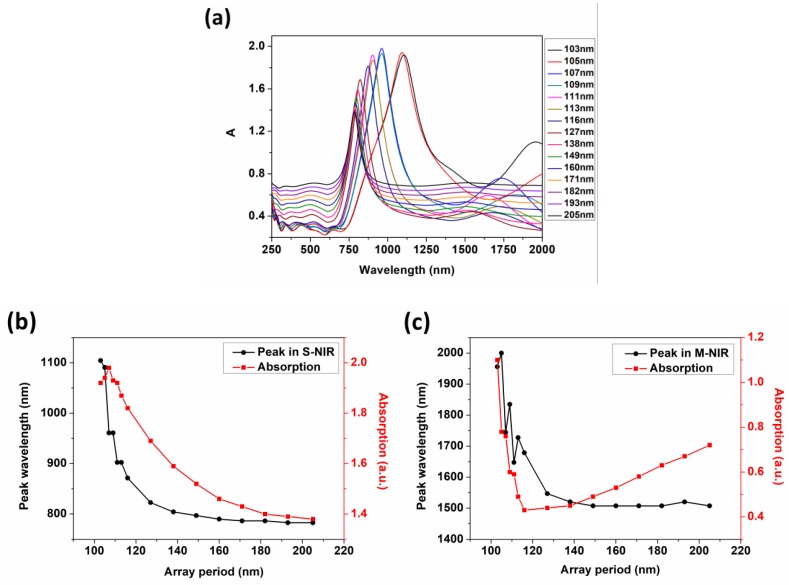
(**a**) The absorption spectra under different periods; (**b**) The scatter diagram of the absorption peak positions and intensities in S-NIR; (**c**) The scatter diagram of the absorption peak positions and intensities in M-NIR.

The gap size in array units could be changed under different period, which also led to the vary of absorption strength and the shift of peak [35]. Here we simulated and analyzed the absorption of arrays from visible band to S-NIR and M-NIR. The positions and intensities of absorption peaks are given in Table S1 and Figure 2.

From Figure 2a,b, the peak absorptions increase slightly from 1.92 to 1.98 as the period increases from 103 to 205 nm, then attenuates to 1.38 when the positions of the optical absorption peaks shift from 104 nm to 782 nm in the S-NIR band. This is consistent with the blue shift effect mentioned in [35]. Shifts are due to three resonance modes that exist in the nanostructure, namely ENZ mode (λ_ENZ_), GR mode (λ_GAP_) and LSPR mode (λ_LSPR_). The hybrid resonance modes produced by their interaction can be expressed by the following formula:

(1) $$\lambda_{HCR}=A\lambda_{ENZ}+B\lambda_{GAP}+C\lambda_{LSPR}$$

We define A, B and C as the coupling coefficients. The modes can not only be coupled with a strong mode but also be mutually exclusive (A, B, C can be positive or negative). In the optimal coupling state, the range of absorption regulating can be broadened. When a = 103 nm, the arrays exhibit strong optical absorption intensities from S-NIR to M-NIR owing to the great GR coupling effect caused by the small gap distance. Light was confined within the structure, transmitting and dissipating in the form of SP waves. The blue-shifts indicate the mistuning and splitting occurring between $\lambda_{ENZ}$, $\lambda_{LSPR}$ and $\lambda_{GAP}$, resulting from the gap increase. The effects of GR mode are getting smaller and smaller until it disappears following with the weakening of $\lambda_{HCR}$. At this point, the absorptions are reduced to mainly depend on $\lambda_{ENZ}$ and $\lambda_{LSPR}$.

In addition, the absorption intensity and peak values change greatly when the period *a* increases from 103 to 138 nm, but the trend becomes slow from 138 to 205 nm and finally reaches a steady-state. It also illustrates that the GR effects begin to disappear as the period increases. Meanwhile, the $\lambda_{HCR}$ is only influenced by $\lambda_{ENZ}$ and $\lambda_{LSPR}$ and the absorption has a tendency to a stable value.

Also we can see from Figure 2, the drifts of optical absorptions and the changes of absorption intensities in S-NIR band are relatively smooth, but they are different in the M-NIR band. In Figure 2c, the fluctuation of the peak positions can be explained as the coupling and exclusion between $\lambda_{ENZ}$, $\lambda_{LSPR}$ and $\lambda_{GAP}$ as the period *a* is small. However, when *a* is larger, the absorption peak positions also tend to a stable value, but the absorption intensity increases slightly. This is caused by the weakening and disappearance of the effects of $\lambda_{GAP}$ and the absorptions in the M-NIR band when the period increases to a large extent. In the meantime, the interaction between light and arrays is left to the coherent absorption near that of the SiO_2_/Cu/ITO multilayers, which is similar to the results in Figure 6.

**Figure 3:**
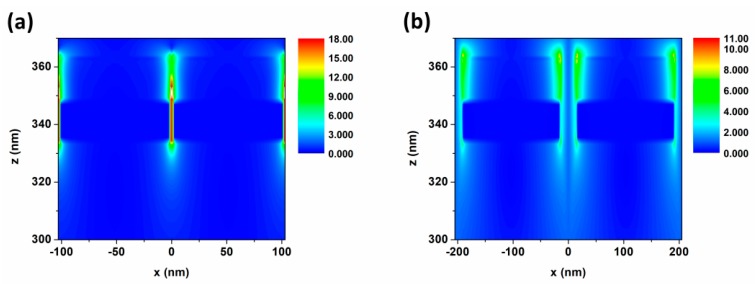
Cross-sectional electric field distributions of arrays with period a = 103 nm (**a**); and 205 nm (**b**).

To verify the disappearance of gap coupling resonance, we calculated the distributions of gap electric field in arrays as shown in Figure 3 and Figure S2. From *a* = 103 nm to 205 nm, the GR mode disappears and the electric field intensity decreases. The variation of electric field distribution is in agreement with the conclusion of optical absorption. Therefore, the blue shift of optical resonance absorption peaks in S-NIR band from 1104 nm to 782 nm and the blue shift of M-NIR band from 1995 nm to 1507 nm was realized under the coupling and detuning of $\lambda_{ENZ}$, $\lambda_{LSPR}$ and $\lambda_{GAP}$ with the period increasing from 103 to 205 nm. The manipulation of the optical absorption in S-NIR and M-NIR bands was achieved between 1.1 to 0.72 and 1.92 to 1.38 concurrently.

### 3.2. Modulation of Absorption by Layer Thickness

**Figure 4:**
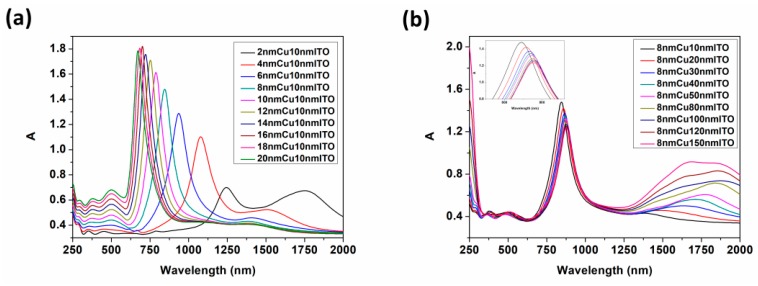
Regulation of the absorption under different layer thicknesses: (**a**) shows the case of different Cu thicknesses; (**b**) shows the case of different ITO thicknesses.

To investigate the effect of layer thickness on HCR mode and the regulation of the absorption in the array, we simulated the optical absorptions with different thicknesses of Cu and ITO (Figures S3–S6, Table S2). The absorption spectra under 10 nm ITO and 8 nm Cu conditions are shown in Figure 4.

From Figure 4a, the spectral drift can be obtained from the S-NIR band. As the Cu layer thickens, the absorption peaks shift to blue and the absorption intensities increase uniformly. Firstly, as the thickness increases, the collision between photons and metal atoms is intensified which causes the absorption of light. Secondly, the height-width ratio of the gap is added with the thickness, which results in the enhancement of the GR effect ($\lambda_{GAP}$). Thirdly, according to the following formula [45]:

(2) $$\omega_{p}{}^{2}=\frac{ne^{2}}{\varepsilon_{0}m^{*}}$$

where, ε_0_ is the dielectric constant in free space, *n* is the carrier concentration, *e* is the electron charge, and m* is the effective mass of the electron. With the increase of the thickness, the free carrier concentration and the plasma oscillation frequency increase which cause the reduction of $\lambda_{LSPR}$. However, when the film is too thick, the LSPR effect is weakened but the effects of GR are more obvious (Figure 7). Thus, the overall coupling effect in the arrays is strengthened, and the absorption peaks show a uniform blue shift in S-NIR band. In the M-NIR band, the absorption peaks shifts slightly to blue with the increase of the thickness when ITO is thin, but with the increase of the thickness of Cu layer, the changes of absorptions are not evidently (Figure S5c).

Compared with Figure 4a, the main variation of absorptions in Figure 4b is gathered around the M-NIR band. With the increase of ITO thickness, the absorptions in M-NIR band show a red-shift enhancement trend which can be explained in the first instance by the Mie theory [46,47,48]:

(3) $$E\left(\lambda \right)=\frac{24\pi^{3}Na^{3}\varepsilon_{m}{}^{3/2}}{\lambda \ln\left(10\right)}\left[\frac{\varepsilon_{i}}{{\left(\varepsilon_{r}+\chi \varepsilon_{m}\right)}^{2}+\varepsilon_{i}{}^{2}}\right]$$

Here, E(λ) is the extinction spectrum, χ is the shape factor, $\varepsilon_{m}$ is external dielectric constant, $\varepsilon_{r}$ is real metal dielectric constant and $\varepsilon_{i}$ is imaginary metal dielectric constant. We analogize the unit arrays to nanoparticles. Therefore, $\lambda_{LSPR}$ increases with the rising “diameter” [41]. Similarly, the LSPR effect is valid near the tip of the interface as the ITO layer is too thick. In this case, the gap coupling effect ($\lambda_{GAP}$) undergoes an enhancement (Figure 7). Secondly, the ENZ mode of ITO ($\lambda_{ENZ}$) can be stimulated and effectively restrict the optical wave to propagate and dissipate along the ENZ layer in the M-NIR band (Figure 6), so under the combined effect ($\lambda_{HCR}$), the resonance peaks in M-NIR band show red-shifts and enhancement with the increase of ITO thickness. However, in the S-NIR band, the absorption peaks shift to red and the absorption intensities decrease slightly. For these reasons, the distance between Cu and the surface layer was increased which reduced the LSPR effect, resulting in the weakening of the HCR effect in the S-NIR band.

**Figure 5:**
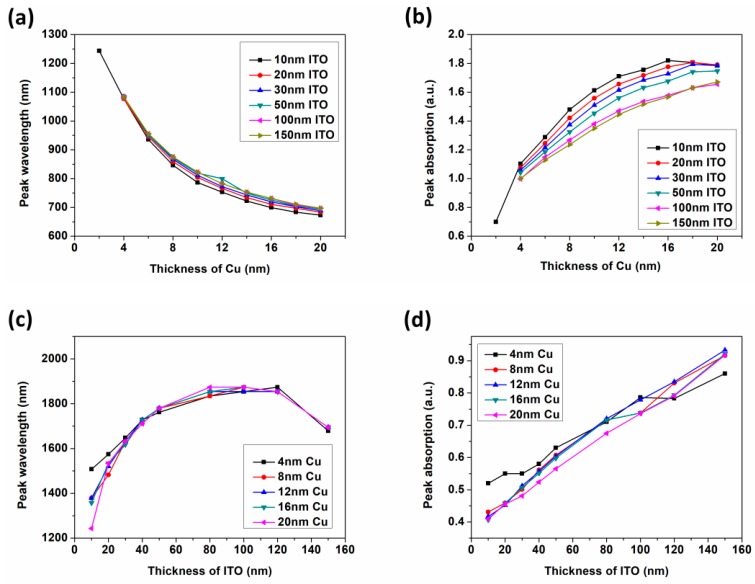
Scatter diagrams of absorption peak positions and absorption intensities of SiO_2_/Cu/ITO arrays, (**a**) peak wavelengths with different Cu thicknesses in S-NIR; (**b**) peak intensities with different Cu thicknesses in S-NIR; (**c**) peak wavelengths with different ITO thicknesses in M-NIR; (**d**) peak intensities with different ITO thicknesses in M-NIR.

Therefore, the ITO layer dominates the optical absorption in M-NIR band while the absorption of S-NIR band is mainly affected by the Cu layer. Furthermore, the data of the absorption peaks in the S-NIR band with different thicknesses of Cu (Figure 5a,b) and the M-NIR band with different thicknesses of ITO (Figure 5c,d) were shifted for comparison in detail.

Figure 5a shows that the optical absorptions in the S-NIR band exhibit an even blue shift with ITO layer thickness ranging from 10 nm to 150 nm while the Cu thickness is from 2 nm to 20 nm. Below 16 nm of Cu, the intensities of absorption peaks rise with thickness (Figure 5b). After 16 nm, the absorptions suffer the impact of ITO thickness a little. When it comes to the ITO case in Figure 5c, the absorption peaks of all arrays with the thickness of Cu layer in the range of 4 nm–20 nm show a red shift trend. However, blue shifts anomalously appear when the thickness of ITO reaches 150 nm, but as we learned from Figure 4b that it is indeed due to the increasing of the overall absorption and intensity in a wide range caused by the strong HCR mode. Figure 5d also reveals the tendency of absorption enhancement. Additionally, the curves in Figure 5c,d are closely distributed with a slight oscillation when compared to those of (a) and (b), which also points that the absorption in M-NIR band is less affected by Cu layer.

### 3.3. Verification of the Hybrid Coupled Resonance Mode 

**Figure 6:**
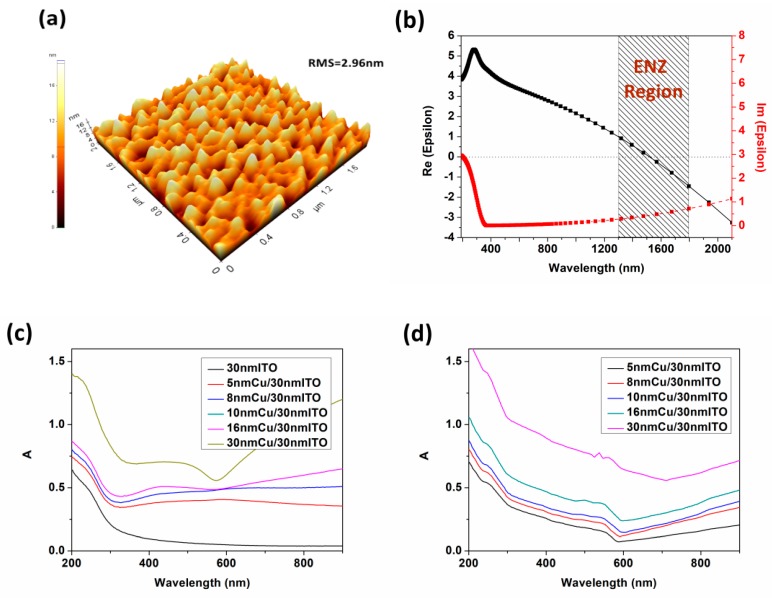
Surface morphology (**a**) and the ENZ region (**b**) of ITO; The absorptions of SiO_2_/Cu/ITO multilayers prepared by experiment (**c**) and calculated by Macleod simulation (**d**).

In order to verify the existence of ITO ENZ mode, LSPR effect and GR coupling, ITO thin films (10 nm) and SiO_2_/Cu/ITO multilayers were prepared. The permittivity of ITO was fitted and analyzed by ellipsometer. The optical absorptions of multilayers were also calculated by TFC Essential Macleod software. From the figure below, the ENZ point of ITO is located at 1526 nm while the ENZ region is spread from 1300 to 1800 nm confirming the existence of ITO ENZ mode in the NIR band. Furthermore, there is no resonance absorption peak in Figure 6c,d compared with the 30 nm ITO condition in Figure S3c. It is conversely proved that the GR and LSPR play a major role in the hybrid resonance mode when no periodic mode exists in the multilayers.

**Figure 7:**
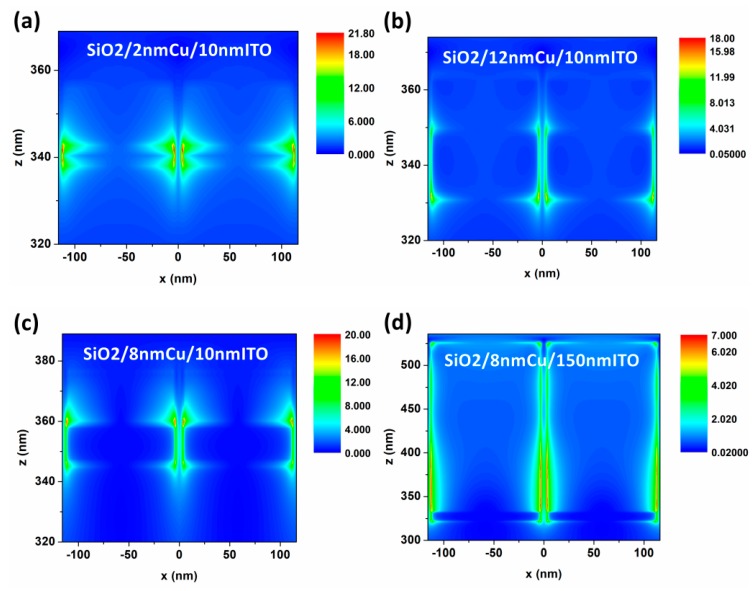
Cross-section electric field distributions of SiO_2_/Cu/ITO microstructures with various wavelengths of incident light: (**a**) 1243 nm; (**b**) 753 nm; (**c**) 1378 nm; (**d**) 1694 nm.

Next, the cross-section distributions of the electric field were analyzed in Figure 7. We have determined that the “hot spots” of the electric field in (a) and (b) are mainly distributed around the Cu layer, while those “hot spots” are majorly on the ITO side in (c) and (d). The conclusion is verified that the absorptions in S-NIR band were mostly modulated by the Cu layer, but are mainly dependent on ITO in M-NIR band. As for the resonant mode, the LSPR effect decreases while the GR mode is more and more widely distributed in the structure when the thickness is large. Finally, the light will be localized in a wider area. Moreover, the electric field distribution conclusion is consistent with the absorptions.

**Figure 8:**
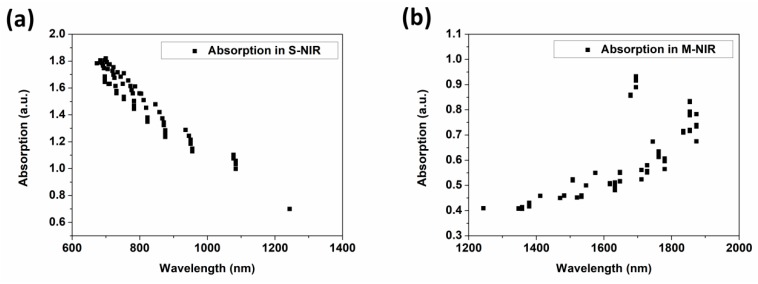
The results of optical absorption peaks modulated by thickness: (**a**) the results in different Cu layers, (**b**) the results of in different ITO layers.

Finally, all optical absorption peaks modulated by thickness are gathered in the form of scatter diagrams in Figure 8. It is absolutely clear that the absorptions are steadily regulated by Cu layers in the S-NIR band, and by ITO layers in the M-NIR band. 

## 4. Conclusions

Based on FDTD simulations, we studied the selective spectral tailoring and manipulation in the broad-spectrum range from the near-infrared short-wave band (S-NIR) to the near-infrared middle-wave band (M-NIR) based on the alteration of periods, Cu and ITO thicknesses in SiO_2_/Cu/ITO arrays. By increasing the period from 103 to 205 nm, the blue shifts of optical absorption peaks from 1104 nm to 782 nm in S-NIR band and from 1995 nm to 1507 nm in M-NIR band were realized. In the meantime, the absorption intensities in S-NIR and M-NIR bands were tuned from 1.1 to 0.72 and from 1.92 to 1.38, respectively. When it comes to the varying thickness of Cu and ITO layers, a steady modulation of the optical absorption peaks from 1243 nm to 673 nm was obtained in the S-NIR band, while in the M-NIR band, they were broadly regulated from 1873 to 1243 nm. The absorption intensities were regulated from 0.69 to 1.80 and from 0.41 to 0.92, respectively, in the S-NIR and M-NIR bands. This tailoring and manipulation originated from the hybrid coupled resonance mechanism which suffered from the co-connection of ENZ mode, LSPR effect and GR coupling. Finally, the absorption modulation and the existence of hybrid mechanism were verified by electric field distributions, film preparation and Macleod calculations. This work is of great significance for the research into selective absorption and spectral modulation of ITO-based metamaterials applied in plasmonic devices in the NIR.

## Supplementary Materials

The following are available online at https://www.mdpi.com/2079-4991/9/6/852/s1.
