# Supplementary material for: Broadband Absorption Tailoring of SiO2/Cu/ITO Arrays Based on Hybrid Coupled Resonance Mode
Source: Nanomaterials (Basel). 2019 Jun 4;9(6):852. doi: 10.3390/nano9060852 (PMC6630435; doi:10.3390/nano9060852)
Supplement: Supplementary file 1 [file nanomaterials-09-00852-s001.pdf]

Supplementary materials

S1 The theory concerned

S1.1 Finite Difference Time Domain Method (FDTD)

Finite-Difference Time-Domain (FDTD) is a commonly used numerical method in electromagnetic field, which uses differential and integral forms. It differentiates Maxwell equation in time and space. The electric field and magnetic field in the frog-jump space domain are calculated alternately, and the change of electromagnetic field is simulated by updating in the time domain to achieve the purpose of numerical calculation.

S1.2 Drude model

Drude-Sommerfeld model is usually used to describe the optical constants of metal and metal-like dielectric films[1]:

$$\varepsilon(\omega) = \varepsilon_1(\omega) + i\varepsilon_2(\omega) = \varepsilon_\infty - \frac{{\omega_p}^2}{\omega^2 + i\Gamma\omega} \quad \textbf{(1)}$$

ε_∞ is the background dielectric constant (high frequency limit), ω_p is the plasma frequency, and Γ represents the charge carrier collision rate, which directly affects the optical loss of materials. The plasma frequency ω_p can be described as:

$${\omega_p}^2 = \frac{ne^2}{\varepsilon_0 m^*} \quad \textbf{(2)}$$

Here, ε_0 is the dielectric constant in free space, n is the carrier concentration, e is the electron charge, and m* is the effective mass of the electron. The change of plasma oscillation frequency will directly affect the optical coupling mode between light and material or microstructures, thus modulating the position and intensity of optical absorption peaks.

S1.3 Surface plasma coupling effect

In optical microstructural arrays, the sizes of structures and materials are in the sub-wavelength range, where metals exhibit extraordinary local surface plasmon resonance (LSPR) effect. Based on Drude model, the LSP oscillation frequencies of lossless dielectrics in dielectrics and Drude metals are obtained as follows：

$$\omega_{LSP} = \frac{\omega_p}{\sqrt{1+2\varepsilon_m}} \tag{3}$$

Here, ω_{LSP} is for SPR peak frequency, ω_p is SPs frequency, the negative dielectric constant of the metal at this time is $\varepsilon_m = n^2$, n is the refractive index of environmental medium. According to reference [2,3]，the resonant wavelength is finally calculated by the relationship between λ_0 and n shown as follows:

$$\lambda_0 = \frac{4\pi c\sqrt{1+\frac{1-L}{L}\varepsilon_d}}{\omega_p} = \lambda_p\sqrt{1+\frac{\frac{1}{L}-1}{n^2}} \tag{4}$$

In this formula，ε_d is the dielectric constant of environmental medium. It shows that the extinction spectrum wavelength is determined by L (the size of metal nanoparticles), n (the refractive index of environmental media) and λ_p (the equivalent wavelength of metal materials).

In addition, the strength and occurrence conditions of LSPR effect will vary following with the change of size and shape of metal nanoparticles, It can be described by Mie-Theory and Gans-Theory [4-6].

Mie-Theory is described as follows:

$$E(\lambda) = \frac{24\pi^3 N a^3 {\varepsilon_m}^{3/2}}{\lambda \, ln(10)}\left[\frac{\varepsilon_i}{(\varepsilon_r+\chi\varepsilon_m)^2+{\varepsilon_i}^2}\right] \tag{5}$$

E(λ) is the extinction spectrum，χ is for shape factor, ε_m is external dielectric constant, ε_r is real metal dielectric constant and ε_i is imaginary metal dielectric constant. The increase of diameter a can lead to the redshifts of the resonance wavelength (λ_{LSPR}).

Gans-Theory extends the influence of shape on material extinction to

nano-ellipsoid and nanorod [7]. The extinction coefficient is expressed as:

$$\sigma_{ext}(\lambda) = \frac{2\pi V \varepsilon_m^{3/2}}{3\lambda} \sum_j \frac{\left(1/P_j^2\right)\varepsilon_i}{\left(\varepsilon_r + \frac{1-P_j}{P_j}\varepsilon_m\right)^2 + (\varepsilon_i)^2} \qquad (A > B = C) \tag{6}$$

Here，V is the spherical particle volume and ε_m is the dielectric constant of the surrounding medium (assumed to be frequency independent).

Among them, the polarization factor is shown as follows:

$$P_A = \frac{1-e^2}{e^2}\left[\frac{1}{2e} ln\left(\frac{1+e}{1-e}\right) - 1\right], \quad P_B = P_C = \frac{1-P_A}{2} \tag{7}$$

$$e = \sqrt{1 - \left(\frac{B}{A}\right)^2} \quad , \quad aspect\, ratio \;\; R = \frac{A}{B} \tag{8}$$

Among them, A represents the long axis of the ellipsoid, B and C are related to the short axis, respectively. There are two maxima in extinction ratio spectra, corresponding to two resonance modes: longitudinal mode and transverse mode.

S1.4 Nonlinear ENZ Mode and Gap Coupled Resonance Mode

When the light is incident on the periodic metal/dielectric optical microstructures, the Epsilon-Near-Zero (ENZ) mode of ITO is excited by the incident light on the film surface. Then, the surface plasmon resonance (SPR) effect is produced with the light passing through ITO film to the metal layer. At the same time, the gap in the structure shows a certain local effect on light. When the light and microstructures are coupled and resonated, the strong electromagnetic field is confined to the gap region between the microstructures. The gap plasma mode is coupled and repulsed with ENZ mode in sub-wavelength microstructures. When they are coupled together, the absorption enhancement and the coupling of light field will be stimulated. When they are mutually exclusive, the coupling mode will be detuned and the mixed coupling mode will split into two separate resonance modes. The ENZ mode has a great influence on the resonance in short near infrared wave, and the gap coupling resonance has a great influence on the long wave range of

near infrared. In addition, the ENZ mode has a very large density of states. In our work, we choose ITO nano-films with sub-wavelength thickness as the top layer, whose near infrared plasma frequency can meet the generation of ENZ mode.

S2 Methods and design

S2.1 Methods

In this paper, SiO_2/Cu/ITO optical microstructures are modeled and simulated by FDTD simulation software (FDTD Solutions of Lumerical Solutions, Canada). The SiO_2/ITO (10 nm) and SiO_2/Cu (10 nm)/ITO (10 nm) thin films were prepared by electron beam evaporation. The surface morphology, permittivity and optical absorption of the films were characterized by atomic force microscopy (XE-100, Park System, Korea), ellipsometer (HORIBA UVISEL-ER, Germany). and ultraviolet-visible-near infrared spectrophotometer (Lambda 1050, Perkin-Elmer, USA). ITO thin films were grown by EB evaporation from ITO coating materials (90 wt. % In_2O_3 and10 wt. % SnO_2 target, 99.99% purity) with baking temperature set at 370°C. The chamber was evacuated to a base pressure of less than 5×10^{-4} Pa. The Cu coating materials was 99.99% pure target. The substrate was 1.35mm quartz glass and the evaporation rate was 0.6Å/s（ITO）and 0.3Å/s（Cu）, respectively. Finally, TFC Essential Macleod was used to calculate the absorption of multilayer films.

S2.2 **FDTD simulation process**

Before FDTD simulating, the original film and structure system were needed to be chosen in advance, and also the relevant material parameters. The specific steps included shape design, material selection, size setting, light source and optical detector addition, scanning setting, mesh addition, optical simulation area setting, material or memory checking, simulation and data

preservation. The following figure shows the material parameters used in the experiment. The ITO data were from our coating experiment.

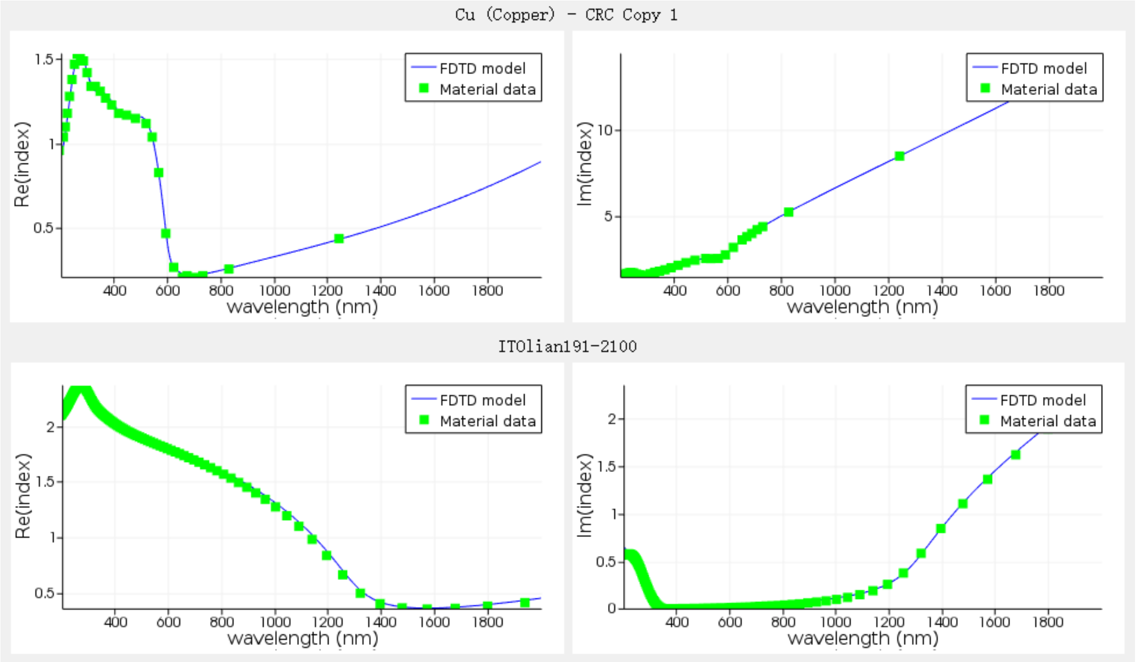

Figure S1. Material index data used in the simulations (including model data and material data of Cu and ITO).

S3 Results and discussions

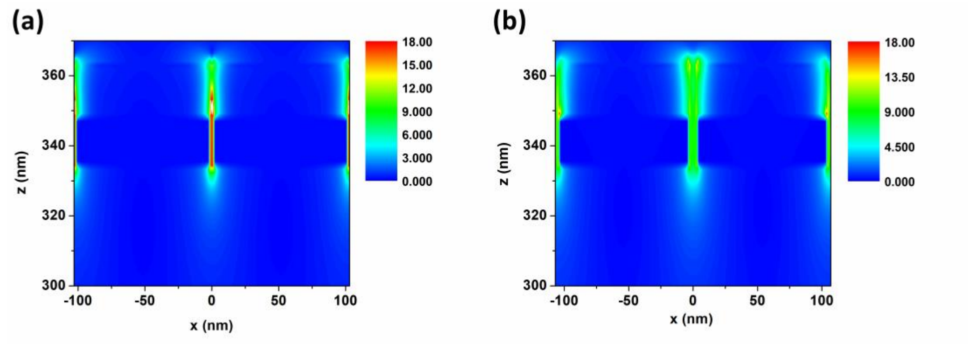

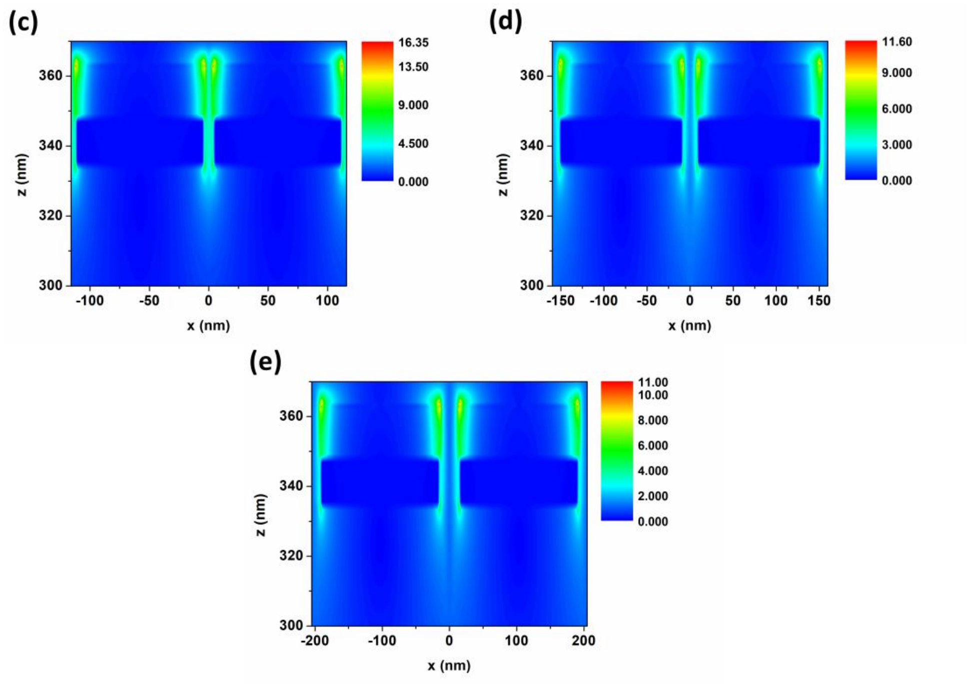

Figure S2. The gap section electric field distribution of microstructures at different periods (a = 103 nm, 107 nm, 116 nm, 160 nm, 205 nm).

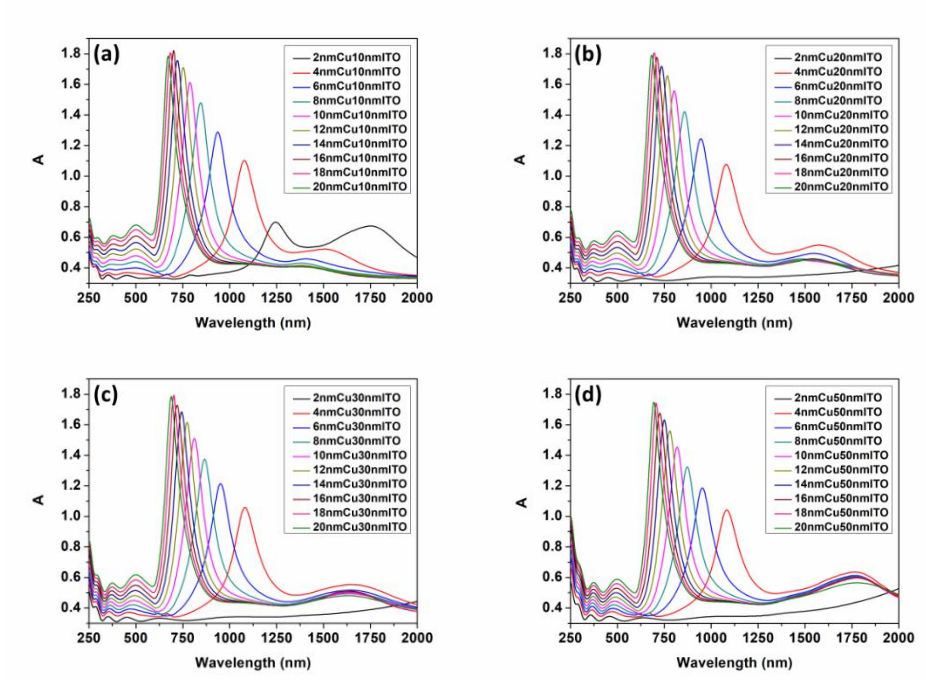

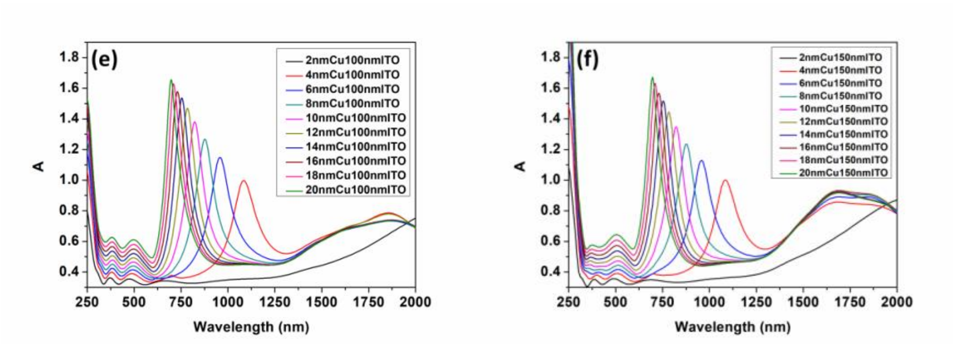

Figure S3. Regulation of absorption by different thicknesses of Cu layers in SiO_2/Cu/ITO microstructures.

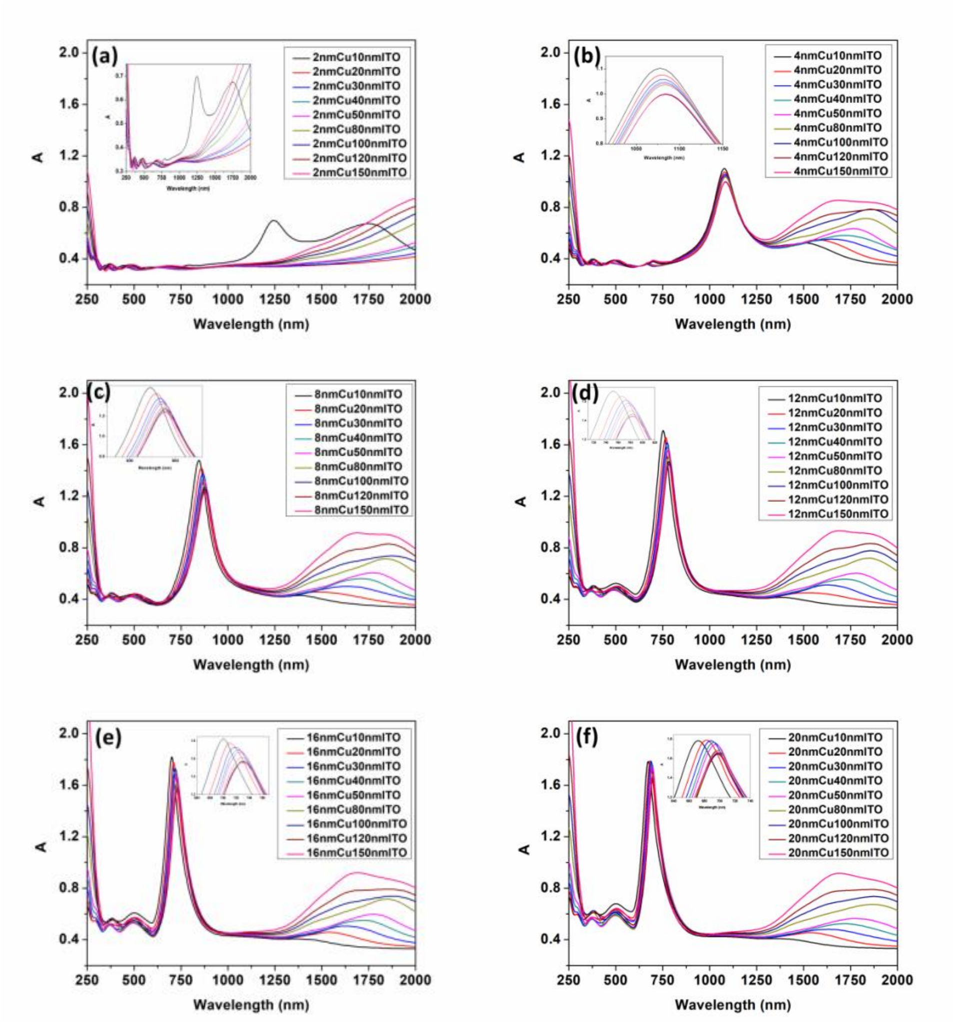

Figure S4. Regulation of absorption by different thicknesses of ITO layers in SiO_2/Cu/ITO microstructures ((a)-(f) represents the case where the thickness of Cu film is 2, 4, 8, 12, 16 and 20 nm, respectively).

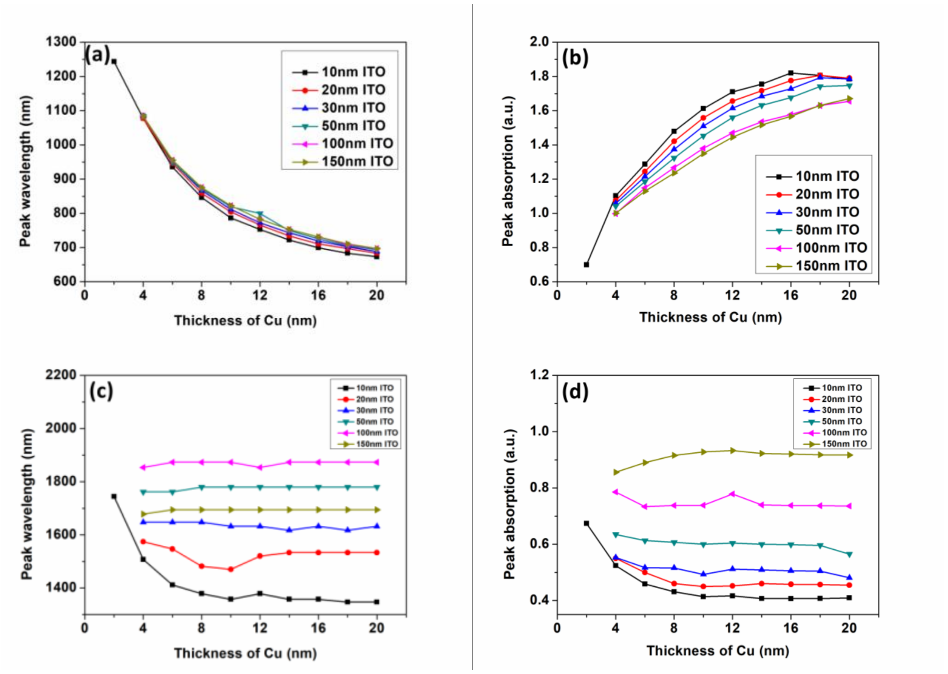

Figure S5. Modulation of absorption peak position and intensity of SiO_2/Cu/ITO microstructures by different thickness of Cu layers ((a) and (b) in S-NIR band, (c) and (d) in M-NIR band).

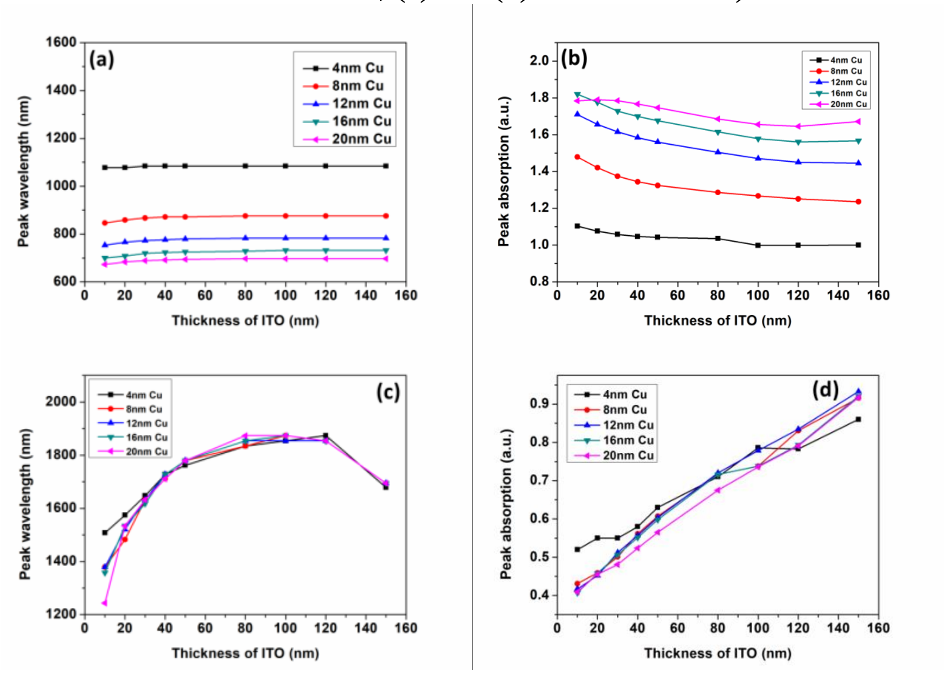

Figure S6. Modulation of absorption peak position and intensity of SiO_2/Cu/ITO microstructures by different thickness of ITO layers ((a) and (b) in S-NIR band, (c) and (d) in M-NIR band)

In order to investigate the influence of the thickness of Cu and ITO on the hybrid coupling resonance mode and the regulation of optical absorption in SiO_2/Cu/ITO arrays, we simulated the optical absorption with different thickness of Cu and ITO, as well as the change of the position and intensity of the corresponding absorption peaks. The thickness of Cu included: 2nm, 4nm, 6nm, 8nm, 10nm, 12nm, 14nm, 16nm, 18nm, 20nm. And the thickness of ITO included 10nm, 20nm, 30nm, 50nm, 100nm and 150 nm. Finally, the position and intensity of absorption peaks in S-NIR and M-NIR bands were obtained as shown in Table S1.

Table S1. The absorption peak location and intensity of the SiO_2/Cu/ITO arrays with different periods in NIR

Samples	a（nm）	Peak in S-NIR（nm）	Absorption of peak（a.u.）	Peak in M-NIR（nm）	Absorption of peak（a.u.）
1	103	1104.35	1.92	1955.94	1.10
2	105	1090.78	1.94	2000.00	0.78
3	107	960.91	1.98	1744.54	0.76
4	109	960.91	1.93	1834.67	0.60
5	111	902.32	1.92	1647.42	0.59
6	113	902.32	1.87	1727.57	0.49
7	116	871.32	1.82	1678.57	0.43
8	127	822.86	1.69	1546.95	0.44
9	138	804.23	1.59	1520.46	0.45
10	149	797.01	1.52	1507.55	0.49
11	160	789.92	1.46	1507.55	0.53
12	171	786.42	1.43	1507.55	0.58
13	182	786.42	1.40	1507.55	0.63
14	193	782.95	1.39	1520.46	0.67
15	205	782.95	1.38	1507.55	0.72

In order to explain the effect of ITO thickness on optical absorption of microstructures in more detail, we simulated the optical absorption of microstructures with ITO thickness as the main variable. The thickness parameters of ITO included: 10nm, 20nm, 30nm, 40nm, 50nm, 80nm, 100nm, 120nm and 150 nm. The thickness of Cu layer was 2nm, 4nm, 8nm, 12nm, 16nm and 20 nm. Finally, the position and intensity of absorption peaks in S-NIR and M-NIR bands were obtained, as shown in Table S2.

Table S2.1 The absorption peak location and intensity of the SiO_2/Cu/ITO arrays with different Cu layer thicknesses in NIR

ITO Thickness（nm）	Cu Thickness（nm）	Peak in S-NIR（nm）	Absorption of peak（a.u.）	Peak in M-NIR（nm）	Absorption of peak（a.u.）
10	2	1243.58	0.69953	1744.54	0.674
10	4	1077.55	1.103	1507.55	0.524
10	6	935.597	1.288	1411.66	0.4586
10	8	846.398	1.479	1378.77	0.43104
10	10	786.417	1.612	1357.69	0.41368
10	12	753.063	1.71	1378.77	0.41653
10	14	722.423	1.755	1357.69	0.40712
10	16	699.65	1.82	1357.69	0.40687
10	18	683.49	1.806	1347.39	0.40736
10	20	673.126	1.784	1347.39	0.40914
20	2	--	--	--	--
20	4	1077.5	1.07619	1574.38	0.55
20	6	945.562	1.244	1546.95	0.5
20	8	858.68	1.421	1482.37	0.46
20	10	804.227	1.558	1470.1	0.45
20	12	766.059	1.656	1520.46	0.452
20	14	734.375	1.717	1533.59	0.46
20	16	710.854	1.776	1533.59	0.458
20	18	696.904	1.806	1533.59	0.457
20	20	683.49	1.79	1533.59	0.455
30	2	--	--	--	--
30	4	1084.12	1.058	1647.42	0.553
30	6	950.625	1.2146	1647.42	0.517
30	8	867.065	1.37407	1647.42	0.516
30	10	811.579	1.51018	1632.28	0.493
30	12	772.727	1.61511	1632.28	0.512
30	14	743.602	1.68403	1617.41	0.509
30	16	719.496	1.728	1632.28	0.506

30	18	702.418	1.793	1617.41	0.505
30	20	688.793	1.78516	1632.28	0.481
50	2	--	--	--	--
50	4	1084.12	1.04215	1761.85	0.635
50	6	950.625	1.185	1761.85	0.613
50	8	871.32	1.3237	1779.51	0.607
50	10	819.06	1.453	1779.51	0.6
50	12	799.512	1.56	1779.51	0.604
50	14	749.883	1.631	1779.51	0.6
50	16	725.374	1.67634	1779.51	0.599
50	18	705.207	1.74136	1779.51	0.596
50	20	694.179	1.747	1779.51	0.565
100	2	--	--	--	--
100	4	1084.12	0.998	1853.83	0.786
100	6	955.742	1.14868	1873.39	0.734
100	8	875.616	1.2672	1873.39	0.738
100	10	822.863	1.3808	1873.39	0.739
100	12	782.95	1.4697	1853.83	0.779
100	14	753.063	1.535	1873.39	0.74
100	16	731.35	1.578	1873.39	0.738
100	18	708.019	1.63	1873.39	0.737
100	20	696.904	1.655	1873.39	0.736
150	2	--	--	--	--
150	4	1084.12	1.0007	1678.57	0.856
150	6	955.742	1.129	1694.59	0.89
150	8	875.616	1.236	1694.59	0.916
150	10	822.863	1.349	1694.59	0.928
150	12	782.95	1.445	1694.59	0.933
150	14	753.063	1.517	1694.59	0.923
150	16	731.35	1.567	1694.59	0.921
150	18	710.854	1.631	1694.59	0.918
150	20	696.904	1.671	1694.59	0.917

Table S2.2 The absorption peak location and intensity of the SiO_2/Cu/ITO arrays with different ITO layer thicknesses in NIR

Cu Thickness（nm）	ITO Thickness（nm）	Peak in S-NIR（nm）	Absorption of peak（a.u.）	Peak in M-NIR（nm）	Absorption of peak（a.u.）
2	10	1243.58	0.69953	1744.54	0.674
2	20	--	--	--	--
2	30	--	--	--	--
2	40	--	--	--	--
2	50	--	--	--	--
2	80	--	--	--	--
2	100	--	--	--	--
2	120	--	--	--	--
2	150	--	--	--	--
4	10	1077.5	1.103	1507.55	0.52
4	20	1077.5	1.076	1574.38	0.55
4	30	1084.12	1.058	1647.42	0.55
4	40	1084.12	1.047	1727.57	0.58
4	50	1084.12	1.042	1761.85	0.63
4	80	1084.12	1.036	1834.67	0.71
4	100	1084.12	0.998	1853.83	0.786
4	120	1084.12	0.9987	1873.39	0.783
4	150	1084.12	1	1678.57	0.86
8	10	846.398	1.479	1378.77	0.431
8	20	858.68	1.4207	1482.37	0.459
8	30	867.07	1.374	1632.28	0.501
8	40	871.32	1.344	1710.9	0.5614
8	50	871.32	1.324	1779.5	0.607
8	80	875.62	1.286	1834.67	0.716
8	100	875.62	1.267	1873.39	0.7378
8	120	875.62	1.251	1853.83	0.8309
8	150	875.62	1.236	1694.59	0.91643
12	10	--	--	--	--

12	20	753.06	1.70997	1378.77	0.417
12	30	766.06	1.656	1520.46	0.452
12	40	772.73	1.615	1632.28	0.512
12	50	776.105	1.584	1727.57	0.557
12	80	779.512	1.56	1779.51	0.6036
12	100	782.95	1.504	1853.83	0.7203
12	120	782.95	1.47	1853.83	0.7787
12	150	782.95	1.45	1853.83	0.8345
16	10	699.65	1.8199	1357.69	0.407
16	20	708.02	1.776	1533.59	0.458
16	30	719.5	1.728	1617.41	0.5062
16	40	722.42	1.699	1727.57	0.5513
16	50	725.37	1.676	1779.51	0.5979
16	80	728.35	1.615	1853.83	0.716
16	100	731.35	1.578	1873.39	0.738
16	120	731.35	1.561	1853.83	0.793
16	150	731.35	1.567	1694.59	0.921
20	10	673.126	1.784	1243.58	0.40975
20	20	683.49	1.79	1533.59	0.455
20	30	688.79	1.7852	1632.28	0.481
20	40	691.48	1.767	1710.92	0.524
20	50	694.179	1.747	1779.51	0.565
20	80	696.904	1.6857	1873.39	0.675
20	100	696.904	1.65541	1873.39	0.736
20	120	696.904	1.6457	1853.83	0.791
20	150	696.904	1.6712	1694.59	0.917

References

1. Kim, J.; Naik, G.V.; Emani, N.K.; Guler, U.; Boltasseva, A. Plasmonic Resonances in Nanostructured Transparent Conducting Oxide Films. *IEEE Journal of Selected Topics in Quantum Electronics* **2013**, *19*, 4601907-4601907.
2. Miller, M.M.; Lazarides, A.A. Sensitivity of metal nanoparticle surface plasmon resonance to the dielectric environment. *Journal Of Physical Chemistry B* **2005**, *109*, 21556-21565, doi:10.1021/jp054227y.
3. Johnson, P.B.; Christy, R.W. Optical Constants of the Noble Metals. *Physical Review B* **1972**, *6*, 4370-4379, doi:10.1103/PhysRevB.6.4370.
4. Mie, G. Beitrage Zur Optik Trüber Medien, Speziell Kolloidaler Metallosungen. *Annalen der Physik* **1908**, *330*, 377-445.
5. Lock, J.A.; Gouesbet, G. Generalized Lorenz-Mie theory and applications. *Journal of Quantitative Spectroscopy & Radiative Transfer* **2009**, *110*, 800-807, doi:10.1016/j.jqsrt.2008.11.013.
6. Mundy, W.C.; Roux, J.A.; Smith, A.M. Mie scattering by spheres in an absorbing medium. *Journal of the Optical Society of America* **1974**, *64*, 1593-1597, doi:10.1364/josa.64.001593.
7. Link, S.; El-Sayed, M.A. Size and Temperature Dependence of the Plasmon Absorption of Colloidal Gold Nanoparticles. *The Journal of Physical Chemistry B* **1999**, *103*, 4212-4217, doi:10.1021/jp984796o.
